# Beyond Life's Essential 8: optimizing cardiovascular health metrics to predict mortality

**DOI:** 10.1016/j.ijcrp.2025.200523

**Published:** 2025-10-02

**Authors:** Yang Peng

**Affiliations:** a Greater Brisbane Clinical School, Faculty of Medicine, The University of Queensland, Brisbane, Australia; b Department of Cardiology, The Prince Charles Hospital, Brisbane, Australia

**Keywords:** Cardiovascular health, Prediction, Mortality

## Abstract

**Background:**

Cardiovascular health (CVH), as assessed by the American Heart Association's Life's Essential 8 (LE8), is strongly associated with mortality risk. However, whether rescoring or weighting individual CVH components improves mortality prediction remains unclear.

**Methods:**

Using data from the 2005–2018 National Health and Nutrition Examination Survey, we examined the associations between CVH categories and risks of all-cause and cardiovascular disease (CVD) mortality. We compared three CVH scoring approaches: the original LE8 model, a rescored model with recalibrated eight metrics, and a weighted model assigning relative importance to each metric. Cox proportional hazards models adjusted for confounders estimated hazard ratios. Model performance was evaluated by C-statistic and net reclassification improvement.

**Results:**

Among 32,076 US adults followed for a median of 7.5 years, higher CVH was consistently associated with lower all-cause and CVD mortality risks across all models. Compared to individuals with low CVH, individuals with high CVH had 58 %–78 % lower all-cause mortality risk and 64 %–87 % lower CVD mortality. For CVD mortality, the rescored model improved risk reclassification, while the weighted model improved discrimination. Compared to the original model, both rescored and weighted models are with modest improvements in all-cause mortality prediction. Weighting revealed substantial variation in the contribution of individual CVH components to mortality risk.

**Conclusions:**

Higher CVH is strongly protective against mortality. Refining LE8 scoring through rescoring and weighting can enhance mortality risk discrimination and reclassification, supporting improved CVH assessment for targeted prevention.

## Highlights

• Higher CVH is linked to lower all-cause and CVD mortality risk.
• Rescoring and weighting CVH improves all-cause mortality prediction.
• Weighting CVH could improve discrimination of CVD mortality prediction.
• Rescoring CVH could improve reclassification of CVD mortality prediction.
• Individual CVH metrics contribute unequally to mortality prediction.

## 1 Introduction

Cardiovascular disease (CVD) remains the leading cause of morbidity and mortality worldwide, accounting for an estimated 20.5 million deaths in 2021 [1]. To address this burden, public health efforts have increasingly focused on identifying and promoting ideal cardiovascular health (CVH). In 2022, the American Heart Association (AHA) updated its CVH metric from "Life's Simple 7" [2] to "Life's Essential 8" (LE8) [3], incorporating sleep health as an eighth component and refining the scoring methodology to improve the assessment of individual and population-level CVH.

LE8 comprises eight modifiable health behaviors and factors: diet, physical activity, nicotine exposure, sleep duration, body mass index (BMI), blood pressure, blood glucose, and blood lipids. Each component is scored on a 0–100 scale, and an unweighted average is used to derive an overall CVH score [3]. While the LE8 was significantly associated with mortality due to all causes and CVD [4], several studies suggested that the associations between metrics and mortality varied significantly [5,6]. The phenomenon raised the question of whether rescoring and weighting all metrics based on their associations with the outcomes could improve the prediction validity of the LE8 score.

In this study, we aim to investigate whether rescoring and weighting the LE8 components can enhance the prediction of CVD and all-cause mortality among the National Health and Nutrition Examination Survey (NHANES) adult participants.

## 2 Methods

This study followed the Transparent Reporting of a Multivariable Prediction Model for Individual Prognosis or Diagnosis reporting guideline [7].

### 2.1 Study population

The NHANES is an ongoing, national, cross-sectional survey with a complex, stratified, and multistage probability sampling design of the non-institutionalized US civilian population. The NHANES was approved by the National Center for Health Statistics Research Ethics Review Board, and written informed consent was obtained from all participants.

As sleep health information was firstly collected in the 2005–2006 NHANES cycle, we used data from the 2005–2018 NHANES cycles. Of the 39,749 adults aged ≥20 years, 7588 and 35 were excluded due to missing LE8 components and potential covariates. A further 50 participants were excluded due to missing mortality follow-up data, leaving 32,076 participants in the study.

### 2.2 Definition of LE8

The LE8 score is composed of four health behaviors, namely, diet, physical activity, nicotine exposure, and sleep duration, along with four health factors, including BMI, blood lipids, blood glucose, and blood pressure. Dietary patterns were evaluated using the Healthy Eating Index 2015 [8], obtained from the participants’ 24-h dietary recall. Self-reported questionnaire was used to obtain information on physical activity, nicotine exposure, sleep patterns, history of diabetes, medication use. Physical examination was employed for the measurement of height, weight, and blood pressure. Blood samples were collected for the assessment of non-high-density lipoprotein (non-HDL) cholesterol, plasma glucose, and hemoglobin A1c. The detailed algorithm utilized for calculating the LE8 scores from the NHANES data has been previously documented [3]. Each of the eight CVH metrics was assigned a score ranging from 0 to 100, with the overall LE8 score calculated as an average of these eight metrics. Participants were categorized into three groups based on their overall LE8 score: high CVH (80–100), moderate CVH (50–79), and low CVH (0-49) [9].

### 2.3 Outcomes

Data from NHANES 2005–2018 were linked to mortality data from the National Death Index death certificate records until December 31, 2019. The outcomes included all-cause mortality and CVD-specific mortality (codes I00-I09, I11, I13, I20-I51, and I60-I69) [5] using the International Classification of Disease Tenth Revision.

### 2.4 Covariates

Based on prior knowledge [10,11], sex, age (continuous), race/ethnicity (Hispanic, non-Hispanic White, non-Hispanic Black, and others), educational level (< high school, high school, some college or associates degree, and college graduate or above), the family poverty income ratio (<1.3, 1.3–2.99, and ≥3), and marital status (married/living with partner, divorced/widowed/separated, and unmarried) were selected as covariates.

### 2.5 Statistical analysis

The basic characteristics were displayed across CVH categories using proportions (categorical) or means (continuous) and compared using Chi-square test (categorical) or unadjusted regression analysis (continuous). The multivariable Cox proportional hazard regression model was used to examine the associations between CVH metrics/categories and mortality due to all causes and CVD and hazard ratios (HRs) and 95 % confidence intervals (CIs) were calculated. No violation of proportional hazard assumption was noticed. Participants were followed up to the deaths or end of 2019, whichever came first.

For the original models, the associations of the overall CVH groups and mortality due to all causes and CVD were examined. For the rescored models, the eight metrics were rescored based on their associations with the two outcomes in the current dataset and there are graded inverse associations between metric scores and mortality. The overall rescored LE8 scores were the average of the eight recalibrated scores. For the weighted models, they were based on the rescored models. For both outcomes, a weight was assigned to each LE8 metric, which obtained via the β coefficient of each metric divided by the sum of the β coefficients of the eight metrics [12]. Covariates were also included in the models, and no significant multicollinearity was found across the eight metrics (Variance Inflation Factors <1.3). The overall weighted LE8 scores were calculated by summing the products of each LE8 component score and its corresponding weight and the overall weighted LE8 scores ranged from 0 to 100.

The discrimination and reclassification abilities were compared across the original, rescored and weighted models. The C-statistic, ranged from 0.5 to 1, was used to estimate discrimination, with high value indicate better model performance. The continuous Net Reclassification Improvement (NRI) was used to measure reclassification performance.

All analyses accounted for the complex survey design of the NHANES using appropriate sample weights, clustering, and stratification. All analyses were performed using Stata version 17.0 (Stata Corporation, College Station, TX, USA) and a two-sided *P* < 0.05 indicating statistically significant.

## 3 Results

Table 1 presents the baseline characteristics of participants across different CVH categories. Individuals in the high CVH group were more likely to be female, younger, non-Hispanic White, have higher levels of education and family income, and be never married (all *P* < 0.001).

**Table 1:** Characteristics of participants by original total CVH categories.

	Low CVH	Moderate CVH	High CVH	*P* value
**Overall, n (%)**^a^	5397 (13.7)	21,332 (66.2)	5347 (20.1)	
**All-cause deaths, n (%)**^a^	1030 (15.7)	2069 (7.3)	172 (2.2)	<0.001
**CVD deaths, n (%)**^a^	316 (4.6)	632 (2.1)	43 (0.5)	<0.001
**Sex, n (%)**^a^				<0.001
Male	2651 (48.0)	10,911 (51.6)	1994 (37.6)	
Female	2746 (52.0)	10,421 (48.4)	3353 (62.4)	
**Age (years), mean (95 % CI)**^a^	54.0 (53.4–54.6)	48.2 (47.7–48.7)	40.1 (39.4–40.9)	<0.001
**Race/ethnicity, n (%)**^a^				<0.001
Hispanic	1297 (13.0)	5536 (14.0)	1271 (13.2)	
Non-Hispanic White	2270 (65.4)	9315 (68.5)	2411 (70.4)	
Non-Hispanic Black	1511 (15.8)	4469 (10.8)	710 (6.6)	
Others	319 (5.8)	2012 (6.6)	955 (9.7)	
**Education level, n (%)**^a^				<0.001
< High school graduate	1945 (26.4)	5170 (15.8)	668 (7.3)	
High school graduate	1447 (30.6)	5215 (25.4)	756 (12.3)	
Some college or associates degree	1457 (30.6)	6549 (32.8)	1553 (27.9)	
College graduate or above	548 (12.4)	4398 (26.0)	2370 (52.5)	
**Family PIR, n (%)**^a^				<0.001
<1.3	2130 (29.8)	6240 (19.7)	1133 (14.0)	
1.3–2.99	2006 (36.5)	7443 (31.7)	1543 (24.4)	
≥3	1261 (33.7)	7649 (48.7)	2671 (61.6)	
**Marital status, n (%)**^a^				<0.001
Married/living with partner	3008 (60.7)	13,011 (64.7)	3286 (64.7)	
Widowed/divorced/separated	1726 (27.2)	4736 (19.1)	592 (9.5)	
Never married	663 (12.1)	3585 (16.2)	1469 (25.8)	
**Diet**				
mean score (95 % CI)^b^	22.1 (21.1–23.1)	37.0 (36.2–37.8)	59.9 (58.7–61.0)	<0.001
**Physical activity**				
mean score (95 % CI)^b^	29.0 (27.3–30.6)	72.8 (71.8–73.8)	95.3 (94.7–95.8)	<0.001
**Nicotine exposure**				
mean score (95 % CI)^b^	38.3 (36.7–39.8)	64.3 (63.4–65.2)	90.6 (89.6–91.5)	<0.001
**Sleep health**				
mean score (95 % CI)^b^	67.5 (66.4–68.6)	83.2 (82.7–83.7)	92.8 (92.2–93.3)	<0.001
**BMI**				
mean score (95 % CI)^b^	33.9 (32.7–35.1)	57.9 (57.1–58.7)	86.0 (85.2–86.7)	<0.001
**Blood lipids**				
mean score (95 % CI)^b^	44.8 (43.6–46.0)	63.3 (62.5–64.0)	85.0 (84.1–86.0)	<0.001
**Blood glucose**				
mean score (95 % CI)^b^	57.5 (56.4–58.6)	78.4 (77.8–78.9)	94.3 (93.8–94.9)	<0.001
**Blood pressure**				
mean score (95 % CI)^b^	40.7 (39.3–42.1)	65.7 (65.0–66.4)	90.4 (89.6–91.1)	<0.001
**LE8**				
mean score (95 % CI)^b^	41.7 (41.4–42.0)	65.3 (65.1–65.5)	86.8 (86.6–87.0)	<0.001

CVH, cardiovascular health; CVD, cardiovascular disease; CI, confidence interval; PIR, poverty income ratio; BMI, body mass index; LE8, Life's Essential 8.

a Unweighted numbers and weighted prevalence.

b Weighted mean and 95 % CI.

Over a median follow-up of 7.5 years (interquartile range: 4.2–10.8 years), a total of 3271 deaths occurred, of which 991 were attributed to CVD. Details of the rescoring process for the eight metrics are provided in Supplementary Table 1. After rescoring, the scores dropped markedly for nicotine exposure and blood lipids (Table 2). Table 3 outlines the procedures used for developing the weighted models.

**Table 2:** Mean scores and 95 % confidence intervals of eight metrics and the overall cardiovascular health before and after rescoring.

**Metrics**	**Original score**	**Rescored score**
Diet	39.5 (38.7–40.4)	39.8 (39.1–40.5)
Physical activity	71.3 (70.4–72.2)	63.9 (63.1–64.7)
Nicotine exposure	66.0 (65.0–66.9)	28.4 (28.1–28.7)
Sleep health	83.0 (82.5–83.5)	86.7 (86.3–87.1)
BMI	60.2 (59.5–61.0)	57.9 (57.3–58.5)
Blood lipids	65.1 (64.5–65.7)	42.9 (42.2–43.6)
Blood glucose	78.7 (78.2–79.2)	78.0 (77.4–78.5)
Blood pressure	67.2 (66.5–67.5)	63.5 (62.9–64.0)
Overall CVH	66.4 (65.9–66.8)	57.6 (57.3–57.9)

BMI, body mass index; CVH, cardiovascular health.

**Table 3:** The weighting process for both all-cause mortality and CVD mortality.

Metrics	All-cause mortality		CVD mortality	
	Coefficient	Weight	Coefficient	Weight
Diet	−0.0018172	0.053	−0.0024307	0.063
Physical activity	−0.0048869	0.141	−0.0045893	0.120
Nicotine exposure	−0.0060971	0.176	−0.0078178	0.204
Sleep health	−0.0040650	0.117	−0.0044291	0.116
BMI	−0.0040299	0.116	−0.0050903	0.133
Blood lipids	−0.0043904	0.127	−0.0009891	0.026
Blood glucose	−0.0055485	0.160	−0.0059005	0.154
Blood pressure	−0.0038068	0.110	−0.0070306	0.184
Sum	−0.0346418	1	−0.0382774	1

CVD, cardiovascular disease; BMI, body mass index.

**Fig. 1:**
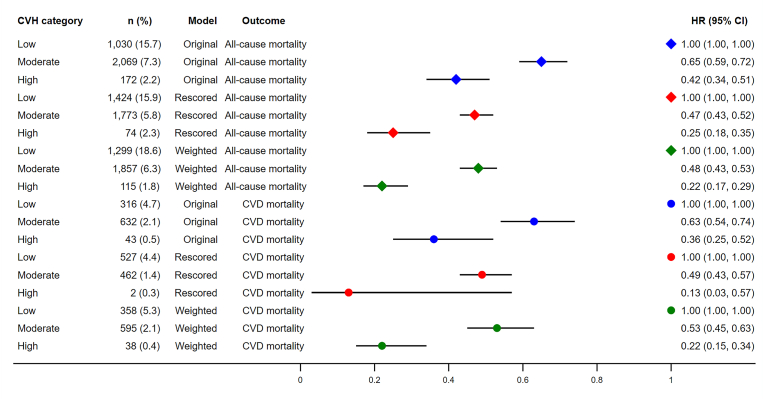
The associations between CVH categories and mortality due to all causes and CVD. CVH, cardiovascular health; CVD, cardiovascular disease; HR, hazard ratio; CI, confidence interval. n (%) were unweighted case numbers and weighted death rates.

In the original model, compared to participants with low CVH, those with moderate and high CVH had a 35 % (HR 0.65, 95 % CI: 0.59–0.72) and 58 % (HR 0.42, 95 % CI: 0.34–0.51) lower risk of all-cause mortality, respectively, after adjusting for potential confounders. These risk reductions increased to 53 % (HR 0.47, 95 % CI: 0.43–0.52) and 75 % (HR 0.25, 95 % CI: 0.18–0.35) in the rescored model, and to 52 % (HR 0.48, 95 % CI: 0.43–0.53) and 78 % (HR 0.22, 95 % CI: 0.17–0.29) in the weighted model (Fig. 1).

The C-statistics for all-cause mortality prediction were 0.856 (95 % CI: 0.850–0.863) for the original model, 0.861 (95 % CI: 0.855–0.867) for the rescored model, and 0.861 (95 % CI: 0.855–0.868) for the weighted model. Both the rescored and weighted models demonstrated significantly improved discrimination compared to the original model (*P* < 0.001). However, the difference between the rescored and weighted models was not statistically significant (*P* = 0.58). Improvements in risk reclassification were also observed for the rescored and weighted models relative to the original model, with NRI and 95 % CI values of 0.227 (0.227–0.227) and 0.201 (0.201–0.201), respectively. When comparing the weighted model to the rescored model, the NRI (95 % CI) was 0.223 (0.223–0.223).

For CVD mortality, participants with moderate and high CVH in the original model had a 37 % (HR 0.63, 95 % CI: 0.54–0.74) and 64 % (HR 0.36, 95 % CI: 0.25–0.52) lower risk, respectively, compared to those with low CVH. These reductions were greater in the rescored model, 51 % (HR 0.49, 95 % CI: 0.43–0.57) and 87 % (HR 0.13, 95 % CI: 0.03–0.57), and in the weighted model, 47 % (HR 0.53, 95 % CI: 0.45–0.63) and 78 % (HR 0.22, 95 % CI: 0.15–0.34) (Fig. 1).

The C-statistics for CVD mortality prediction were 0.887 (95 % CI: 0.878–0.896) for the original model, 0.889 (95 % CI: 0.879–0.898) for the rescored model, and 0.889 (95 % CI: 0.880–0.898) for the weighted model. Compared to the original model, a significant improvement in discrimination was noticed in the weighted model (*P* = 0.01) instead of the rescored model (*P* = 0.13). No difference was observed between the rescored and the weighted model (*P* = 0.58). The rescored model showed improved reclassification compared to the original model, with an NRI of 0.082 (95 % CI: 0.082–0.082). In contrast, the weighted model demonstrated poorer reclassification performance, with an NRI of −0.009 (95 % CI: −0.009 to −0.009) compared to the original model. It has improved reclassification with an NRI of 0.212 (95 % CI: 0.212–0.212) compared to the rescored model.

## 4 Discussion

In this study, we examined the associations between CVH categories and the risks of all-cause and CVD mortality. The rescoring and weighting of each metric could have a greater impact on mortality reduction. They could modestly improve the predictive performance of risk models, especially for all-cause mortality.

Consistent with previous studies [5,6], participants with moderate or high CVH had significantly lower risks of both all-cause and CVD mortality compared to those with low CVH. These inverse associations were more pronounced when using the rescored and weighted models, suggesting that updated CVH metrics may better capture the protective effects of favorable health profiles. Notably, the weighted model, which reflects the relative importance of individual CVH components, yielded the greatest reduction in all-cause mortality among individuals with high CVH. In contrast, a pooled analysis of two large US cohort indicates that weighting has limited effects on the combined associations of BMI and lifestyle factors, including diet, physical activity, alcohol consumption, and smoking, with all cause and cardiovascular mortality [13].

To the best of our knowledge, this is the first study to evaluate whether rescoring or weighting the LE8 metrics could improve mortality prediction. Model discrimination, assessed by the C-statistic, was high (>0.85) across all models, particularly for CVD mortality. It is interesting to observe that rescoring and weighting could further improve the model discrimination for all-cause mortality. As the absolute differences were quite small, and the clinical impact may be limited. For CVD mortality, no significant improvement of discrimination was observed for the rescored model. This may reflect a ceiling effect, given the already strong performance of the original model in predicting CVD mortality (C-statistic = 0.887). Modest improvement in risk reclassification further supported the advantages of refining CVH metrics. Both rescored and weighted models showed improved reclassification for all-cause mortality. However, for CVD mortality, only the rescored model led to modest improvements, while the weighted model demonstrated poorer performance. This discrepancy suggests that although weighting may enhance general mortality prediction, it may not improve, and could even impair, risk reclassification for CVD mortality. One possible explanation is that weighting may inadvertently place greater emphasis on metrics less directly related to CVD mortality, while underrepresenting the influence of key cardiovascular risk factors.

The scores changed dramatically for BMI, non-HDL cholesterol and blood pressure in the rescoring process. Recent studies have also highlighted non-linear (e.g., J-shaped or U-shaped) associations between BMI, non-HDL cholesterol, blood pressure and both all-cause and CVD mortality [[14], [15], [16], [17]], indicating that both low and high levels of these markers were associated with increased risk. Our findings challenge the prevailing “lower is always better” paradigm, as seen in recent literature. For example, BMI below 23 kg/m^2^ in older adults has been linked to increased mortality risk [18]. Similarly, weight loss trajectories in mid‐ to late life (including unintentional weight loss) are warning signs for adverse outcomes including mortality [19]. In the realm of blood pressure, the Cochrane review indicates that using very aggressive blood pressure targets in older adults may not reduce mortality and may risk harms from lowered perfusion or adverse effects [20]. Lipid lowering in older, frail persons is also increasingly approached with nuance: guidelines and expert reviews recommend tailoring treatment intensity to life expectancy, frailty, comorbidity, not just risk factor levels [21]. These findings suggest that the AHA should consider revising the ideal thresholds for the three metrics in future updates to the LE8 framework.

The weighting analysis further suggests that the contributions of the eight CVH metrics to mortality vary substantially. For instance, nicotine exposure, assigned the highest weight (0.176 and 0.204), was the strongest predictor of all-cause and CVD mortality, whereas diet had the lowest weight for all-cause mortality (0.053) and second lowest weight for CVD mortality (0.063). These results suggest that equal weighting of CVH metrics may obscure important differences in their individual predictive contributions and could lead to suboptimal risk stratification.

This study has several strengths, including the use of a large, nationally representative sample, comprehensive adjustment for confounding variables, and the application of advanced risk modelling and reclassification methods. However, several limitations should be acknowledged. First, due to its observational design, causal inferences cannot be made. Second, CVH metrics were measured only at baseline, and changes over time were not accounted for. Third, the weighting scheme was derived empirically from the current dataset and requires external validation before clinical application. Fourth, it is based on US general population and may not applied to other population. Fifth, the follow up is relative short to capture deaths. Moreover, we are unable to explore LE8 and CVD incidence because NHANES does not have the outcome data.

## 5 Conclusion

Higher CVH is strongly associated with lower risks of all-cause and CVD mortality. Refinement of CVH scoring through metric rescoring and weighting can modestly enhance model performance while the clinical implication needs further exploration. These findings support ongoing efforts to improve CVH assessment tools to better identify at-risk individuals and guide targeted prevention strategies.

## Funding

None declared.

## Declaration of competing interest

None declared.
