# Supplementary material for: Beyond Life's Essential 8: optimizing cardiovascular health metrics to predict mortality
Source: Int J Cardiol Cardiovasc Risk Prev. 2025 Oct 2;27:200523. doi: 10.1016/j.ijcrp.2025.200523 (PMC12528943; doi:10.1016/j.ijcrp.2025.200523)
Supplement: Multimedia component 1 [file mmc1.docx]

**Supplementary Table 1** The rescoring process of the metrics.

| **Original score** | **All-cause mortality** | | **CVD mortality** | |
| --- | --- | --- | --- | --- |
|  | **HR (95% CI)** | **Rescored score** | **HR (95% CI)** | **Rescored score** |
| **Diet** |  |  |  |  |
| 0 | Reference | 0 | Reference | 50 |
| 25 | 0.99 (0.87-1.14) | 25 | 1.05 (0.81-1.37) | 0 |
| 50 | 0.91 (0.81-1.02) | 50 | 1.00 (0.78-1.30) | 25 |
| 80 | 0.73 (0.63-0.84) | 80 | 0.81 (0.62-1.06) | 80 |
| 100 | 0.72 (0.60-0.88) | 100 | 0.75 (0.50-1.11) | 100 |
| **Physical activity** |  |  |  |  |
| 0 | Reference | 0 | Reference | 0 |
| 20 | 0.64 (0.45-0.91) | 60 | 0.62 (0.36-1.08) | 80 |
| 40 | 0.54 (0.43-0.68) | 100 | 0.54 (0.34-0.88) | 100 |
| 60 | 0.71 (0.55-0.91) | 40 | 0.70 (0.47-1.03) | 20 |
| 80 | 0.60 (0.46-0.78) | 80 | 0.69 (0.45-1.06) | 60 |
| 90 | 0.73 (0.61-0.89) | 20 | 0.70 (0.51-0.95) | 40 |
| 100 | 0.58 (0.52-0.65) | 90 | 0.59 (0.49-0.72) | 90 |
| **Smoke** |  |  |  |  |
| 0 | Reference | 0 | Reference | 0 |
| 5 | 0.74 (0.34-1.65) | 25 | 0.41 (0.05-3.44) | 100 |
| 25 | 0.84 (0.59-1.20) | 5 | 0.94 (0.43-2.05) | 5 |
| 30 | 0.68 (0.52-0.87) | 30 | 0.47 (0.26-0.83) | 80 |
| 50 | 0.57 (0.50-0.65) | 50 | 0.64 (0.49-0.82) | 50 |
| 80 | 0.54 (0.42-0.70) | 80 | 0.72 (0.48-1.06) | 25 |
| 100 | 0.47 (0.42-0.53) | 100 | 0.68 (0.54-0.86) | 30 |
| **Sleep** |  |  |  |  |
| 0 | Reference | 0 | Reference | 0 |
| 20 | 0.77 (0.52-1.13) | 40 | 0.96 (0.50-1.87) | 20 |
| 40 | 0.78 (0.54-1.12) | 20 | 0.87 (0.49-1.53) | 70 |
| 70 | 0.55 (0.39-0.80) | 70 | 0.73 (0.40-1.33) | 90 |
| 90 | 0.55 (0.37-0.81) | 90 | 0.88 (0.47-1.65) | 40 |
| 100 | 0.52 (0.36-0.75) | 100 | 0.63 (0.35-1.12) | 100 |
| **BMI** |  |  |  |  |
| 0 | Reference | 0 | Reference | 0 |
| 15 | 0.67 (0.53-0.84) | 30 | 0.56 (0.36-0.90) | 15 |
| 30 | 0.53 (0.44-0.65) | 70 | 0.44 (0.31-0.64) | 70 |
| 70 | 0.51 (0.43-0.60) | 100 | 0.36 (0.26-0.51) | 100 |
| 100 | 0.70 (0.59-0.84) | 15 | 0.46 (0.32-0.65) | 30 |
| **Lipids** |  |  |  |  |
| 0 | Reference | 20 | Reference | 40 |
| 20 | 0.81 (0.65-1.01) | 100 | 0.92 (0.64-1.33) | 100 |
| 40 | 0.91 (0.75-1.11) | 60 | 0.96 (0.68-1.35) | 60 |
| 60 | 0.87 (0.70-1.08) | 80 | 0.93 (0.68-1.27) | 80 |
| 80 | 0.98 (0.79-1.20) | 40 | 1.01 (0.74-1.39) | 20 |
| 100 | 1.27 (1.05-1.53) | 0 | 1.03 (0.76-1.40) | 0 |
| **Glucose** |  |  |  |  |
| 0 | Reference | 0 | Reference | 0 |
| 10 | 0.66 (0.44-1.00) | 20 | 0.87 (0.36-2.11) | 10 |
| 20 | 0.74 (0.52-1.06) | 10 | 0.45 (0.21-0.96) | 40 |
| 30 | 0.65 (0.47-0.89) | 30 | 0.55 (0.26-1.16) | 20 |
| 40 | 0.58 (0.42-0.79) | 40 | 0.52 (0.26-1.02) | 30 |
| 60 | 0.38 (0.29-0.51) | 60 | 0.33 (0.17-0.63) | 60 |
| 100 | 0.38 (0.28-0.51) | 100 | 0.31 (0.16-0.59) | 100 |
| **Blood pressure** |  |  |  |  |
| 0 | Reference | 0 | Reference | 0 |
| 5 | 0.91 (0.79-1.06) | 5 | 0.98 (0.77-1.26) | 5 |
| 25 | 0.78 (0.66-0.92) | 50 | 0.70 (0.52-0.95) | 55 |
| 30 | 0.80 (0.67-0.95) | 30 | 0.82 (0.63-1.06) | 30 |
| 50 | 0.60 (0.50-0.71) | 100 | 0.50 (0.38-0.66) | 80 |
| 55 | 0.74 (0.63-0.87) | 55 | 0.83 (0.59-1.17) | 25 |
| 75 | 0.66 (0.54-0.80) | 80 | 0.41 (0.29-0.58) | 100 |
| 80 | 0.87 (0.73-1.03) | 25 | 0.76 (0.56-1.03) | 50 |
| 100 | 0.68 (0.57-0.81) | 75 | 0.59 (0.42-0.84) | 75 |

CVD, cardiovascular disease; HR, hazard ratio; CI, confidence interval; BMI, body mass index.
